# Validating the Capability for Measuring Age-Related Changes in Grip-Force Strength Using a Digital Hand-Held Dynamometer in Healthy Young and Elderly Adults

**DOI:** 10.1155/2020/6936879

**Published:** 2020-04-20

**Authors:** Shu-Chun Lee, Li-Chen Wu, Shang-Lin Chiang, Liang-Hsuan Lu, Chao-Ying Chen, Chia-Huei Lin, Cheng-Hua Ni, Chueh-Ho Lin

**Affiliations:** ^1^School of Gerontology Health Management, College of Nursing, Taipei Medical University, 250 Wu-Xing Street, Taipei 11031, Taiwan; ^2^Department of Nursing, Wan Fang Hospital, 111 Xinglong Rd., Sec. 3, Wenshan Dist., Taipei 11696, Taiwan; ^3^Department of Physical Medicine and Rehabilitation, Tri-Service General Hospital, School of Medicine, National Defense Medical Center, 325 Chenggong Road, Sec. 2, Neihu Dist., Taipei 114xx, Taiwan; ^4^Department of Physical Therapy and Assistive Technology, National Yang-Ming University, 155 Linong Street, Sec.2, Beitou Dist., Taipei 112xx, Taiwan; ^5^Department of Rehabilitation Sciences, The Hong Kong Polytechnic University, ST 531, Core S, 11 Yuk Choi Rd, Hung Hom, Hong Kong; ^6^Department of Nursing, Tri-Service General Hospital Songshan Branch, School of Nursing & School of Medicine, National Defense Medical Center, Taiwan; ^7^Master Program in Long-Term Care & School of Gerontology Health Management, College of Nursing, Taipei Medical University, 250 Wu-Xing Street, Taipei 11031, Taiwan; ^8^Center for Nursing and Healthcare Research in Clinical Practice Application, Wan Fang Hospital, Taipei Medical University, 111 Xinglong Rd., Sec. 3, Wenshan Dist., Taipei 11696, Taiwan

## Abstract

**Background:**

Grip-force performance can be affected by aging, and hand-grip weakness is associated with functional limitations of dasily living. However, using an appropriate digital hand-held dynamometer with continuous hand-grip force data collection shows age-related changes in the quality of hand-grip force control may provide more valuable information for clinical diagnoses rather than merely recording instantaneous maximal hand-grip force in frail elderly adults or people with a disability. Therefore, the purpose of this study was to indicate the construct validity of the digital MicroFET3 dynamometer with Jamar values for maximal grip-force assessments in elderly and young adults and confirmed age-related changes in the maximal and the quality of grip-force performance using the MicroFET3 dynamometer in elderly people.

**Methods:**

Sixty-five healthy young (23.3 ± 4.5 years) and 50 elderly (69.5 ± 5.8 years) adults were recruited and asked to perform a validity test of the grip-force maximum voluntary contraction (MVC) using both the dominant and nondominant hands with a Jamar dynamometer and a MicroFET3 dynamometer.

**Results:**

A strong correlation of maximal grip-force measurements was found between the MicroFET3 dynamometer and Jamar standard dynamometer for both hands in all participants (*p* < 0.05). Although, the results showed that a lower grip force was measured in both hands by the MicroFET3 dynamometer than with the Jamar dynamometer by 49.9%~57% (*p* < 0.05), but confidently conversion formulae were also developed to convert MicroFET3 dynamometer values to equivalent Jamar values for both hands. Both dynamometers indicated age-related declines in the maximum grip-force performance by 36.7%~44.3% (*p* < 0.05). We also found that the maximal hand-grip force values generated in both hand by the elderly adults were slower and more inconsistent than those of the young adults when using the MicroFET3 dynamometer.

**Conclusions:**

This study demonstrated that the digital MicroFET3 dynamometer has good validity when used to measure the maximal grip force of both hands, and conversion formulae were also developed to convert MicroFET3 dynamometer force values to Jamar values in both hands. Comparing with the Jamar dynamometer for measuring grip force, the MicroFET3 dynamometer not only indicated age-related declines in the maximum grip-force performance but also showed slower and more inconsistent maximal hand-grip strength generation by the elderly.

## 1. Introduction

The hand-grip strength performance in both hands plays important roles in daily activities. Aging-induced deterioration of grip strength might occur, and this results in poorer muscle strength and poorer bimanual coordination control by elderly people [1, 2]. Therefore, physical and occupational therapists use clinical evaluation tools to measure the grip strength performance, analyze age-related changes in maximal and submaximal grip-force performances, and determine their impacts on functional performances, which is helpful for developing appropriate exercise programs to improve the maximal grip-force performance of frail elderly people and people with diseases. Additionally, the Asian Working Group for Sarcopenia (AWGS) includes and uses the hand-grip force as an index to set the sarcopenia criterion for sarcopenia screening in elderly adults and indicated that a low muscle mass and lower hand-grip force cause poorer physical performances by community-dwelling older people [3], suggesting that these elderly adults with sarcopenia need exercise programs to improve their hand-grip strength and physical activities. Early studies also pointed out that older adults with sarcopenia are associated with falls and functional limitations and have poor health conditions following an acute illness, including depression and longer hospital stays [2, 4–7]. However, in addition to confirming the decrease in hand-grip strength, identifying unstable hand-grip strength control should be considered as well, because we believe that if an elderly person has sufficient hand-grip strength, but accompanying unstable hand-grip strength control, he or she may not be able to perform daily activities very well. Therefore, physical and occupational therapists also need appropriate measurement tools to evaluate the quality of hand-grip strength control.

Traditionally, the Jamar dynamometer is the gold standard tool for hand-grip strength evaluations with excellent validity and reliability in the clinic and research [8], and many therapists use the Jamar dynamometer for measuring grip force and recording a single maximal or submaximal grip force value during testing [4, 8]. However, the Jamar dynamometer is a mechanical measurement tool and only shows an instantaneous hand-grip force, which means that the Jamar dynamometer cannot continuously record hand-grip force or show changes in the quality of hand-grip force control. The Jamar dynamometer also needs recalibration each year [9], and a recent study reported that its limited contact area may cause hand pain in subjects, thereby influencing the grip-force measurement when applying higher grip strengths [10]. Additionally, the man-made bias of grip strength data recording using the Jamar dynamometer also occurs in the clinic. Therefore, a novel digital dynamometer with automatic calibration, a larger contact area, automatic grip strength data recording, and continuous hand-grip force data collection might be more convenient for therapists for measuring the quality of hand-grip force control. Recently, digital electronic hand-held dynamometers, such as the MicroFET3 dynamometer [11], were developed with excellent validity and reliability and can be used to measure the muscle strength and continuously record changes in the muscle strength performance, improving clinical diagnoses and analyses of the effects of treatments for neuromuscular diseases [12, 13]. The MicroFET3 dynamometer has larger contact areas and can automatically be calibrated before testing for each subject, and data collection can be transferred to a computer via Bluetooth or a USB stick during testing, which is helpful in eliminating man-made bias of data recording [11]. Therefore, the MicroFET3 dynamometer might be more convenient and suitable for evaluating the hand-grip force, but little is known as to whether the MicroFET3 dynamometer can be used to evaluate hand-grip force performance, because its construct validity for measuring the maximal grip force has not been confirmed with the gold standard measurement tool (the Jamar dynamometer). If the MicroFET3 dynamometer can measure grip force as accurately as the Jamar dynamometer, it could then be used clinically to monitor the quality of grip-force control, which would provide valuable information in assisting clinical diagnoses, identifying early signs of potential frailty, and developing appropriate rehabilitation interventions and health promotion programs for frail elderly adults and people with disabilities [14]. Therefore, the purposes of this study were to (1) determine the construct validity of the MicroFET3 dynamometer for maximal grip-force assessments in young and elderly adults in comparison with the Jamar dynamometer and (2) indicate age-related changes in the maximal and the quality of grip-force performance using the MicroFET3 dynamometer in elderly people.

## 2. Materials and Methods

### 2.1. Participants

Sixty-five young (23.3 ± 4.5 years old) and 50 elderly (69.5 ± 5.8 years old) adults were recruited and participated in this study from college and community settings (Table 1). The inclusion criteria included no disease that would impact grip-force generation by the hands, no cognitive impairment, and the ability to follow the researchers' instructions for executing maximal voluntary grip-force contraction tests. Additionally, the elderly adults underwent a Mini-Mental Status Examination, and a minimum score of 24 was required, which was identified as having normal cognitive function [15]. The exclusion criterion was the experience of pain or discomfort during maximal voluntary grip-force contraction tests. This study was approved by the local ethics committee (approval no. N201704083). Each subject gave informed consent before joining the study, and the dominant hand was defined as the one used for writing.

### 2.2. Research Device and Data Processing

The Jamar hand dynamometer (Lafayette Instrument, Lafayette, IN, USA) was used as a gold standard tool to evaluate the maximal voluntary grip-force contraction with a maximum of 90 kg of grip force in 2-kg intervals. The Jamar dynamometer was set at the second handle position to evaluate each participant's grip strength [16]. To perform grip-force measurements, each subject placed the Jamar in their palm and pulled the metal bar toward their palm with their fingers. The MicroFET3 dynamometer (Hoggan Health Industries, Salt Lake City, UT, USA) can measure 68 kg of muscle strength. Data were collected via Bluetooth or a USB stick using the TBS program (vers. 11.0.1) with the sampling rate set to 100 Hz. All grip strength data for the maximal voluntary contraction tests from each subject were shown in real time on a laptop via the TBS program for the clinical evaluator, showing real-time changes in the grip strength.

### 2.3. Experimental Procedures and Positioning

Each participant was comfortably seated and asked to hold the Jamar dynamometer vertically with one hand and execute the maximal voluntary grip-force contraction test. The measurement was then repeated with the other hand after a 30-minute rest period to prevent muscle fatigue [17]. Dominant and nondominant hands were tested in a counterbalanced order, and the grip force was measured with the MicroFET3 and Jamar dynamometers in a counterbalanced order as well. The test position for grip strength for each subject was set with the shoulder adducted and placed in neutral rotation with the elbow joint in 90° flexion, the forearm in a neutral position, and the wrist positioned between 0° and 30° extension according to the recommendations of the American Society of Hand Therapists [18].

### 2.4. Validity Testing

The concurrent validity was examined by comparing the maximal grip forces in kilograms recorded by the MicroFET3 hand-held dynamometer and Jamar. The maximal voluntary grip-force contraction test was used to determine the muscle strength of both hands in each subject. The test was performed by asking a subject to, respectively, grasp the Jamar and MicroFET3 hand-held dynamometers with each hand and generate the maximum grip force three times for a period of 6 seconds each [19]. These repetitions were executed with 60-second rest periods in between to prevent muscle fatigue [20]. A maximal voluntary contraction was defined as the average maximal voluntary contraction value from the three trials [21].

### 2.5. Statistical Analysis

Bland-Altman plots were constructed to examine the difference (bias) between the two dynamometers against the average of dynamometers in both hands for all participants. Horizontal lines were drawn at the mean difference and at the limits of an agreement which were defined as the mean difference ± 1.96 standard deviations (SDs) of differences. Pearson's correlation was used to validate the validity of the maximal grip-force assessment of both hands obtained using the Jamar and MicroFET3 hand-held dynamometers in young and elderly groups. A correlation coefficient (*r* value) of 1 indicates a perfect correlation while 0 indicates no correlation. It was assumed that an *r* value <0.3 represented a negligible correlation, 0.3–0.5 a low correlation, 0.5–0.7 a moderate correlation, 0.7–0.9 a strong correlation, and >0.9 a very strong correlation [22]. Additionally, a two-way analysis of variance (ANOVA) was also used to confirm differences in grip-force performances for both hands between the hand-held dynamometers and groups. The SPSS vers. 17.0 statistical software was used (SPSS, Chicago, IL, USA). The alpha level of statistical significance was set to 0.05. In addition, we pooled all grip force data, collected with the MicroFET3 during validity testing for each subject, were pooled into SigmaPlot software (vers. 10.0, Systat Software Inc, San Jose City, CA, USA). This allowed us to create schematic diagrams that could provide a visual comparison of age-related changes in the patterns of maximal grip force generation for young and elderly adults.

## 3. Results

**Figure 1:**
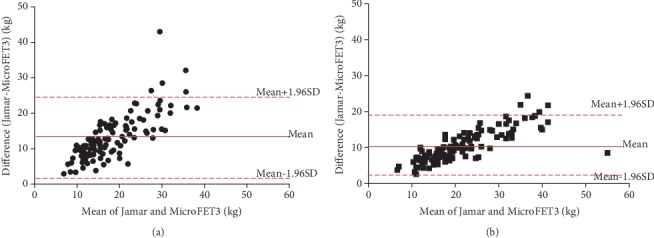
Bland-Altman plot of the mean and difference between the Jamar and MicroFET3 dynamometers for the (a) dominant and (b) nondominant hands in all participants. Biases were 13 kg in the dominant hand and 10 kg in the nondominant hand.

**Figure 2:**
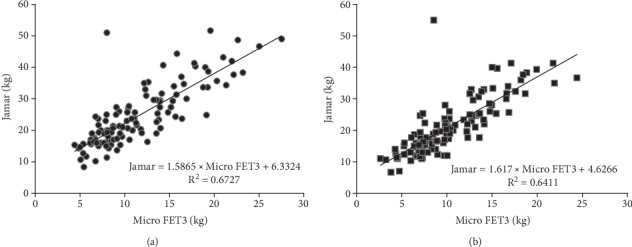
A scatter-plot diagram showing a strong correlation between the MicroFET3 and Jamar dynamometers in (a) the dominant and (b) nondominant hands with formulae to convert MicroFET3 values to equivalent Jamar values.

**Figure 3:**
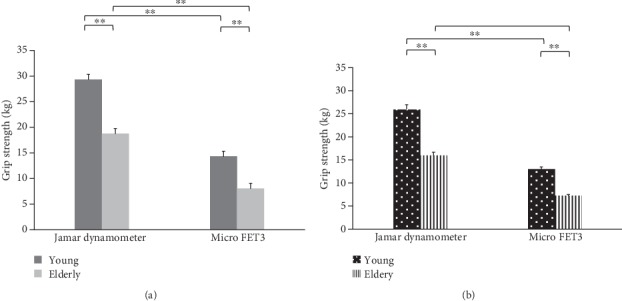
Greater grip force was shown by the Jamar than by the MicroFET3 dynamometer, and a greater amount of maximal voluntary grip-force contraction was shown for young than for elderly adults for (a) the dominant and (b) nondominant hands. ∗*p* < 0.05, ∗∗*p* < 0.001.

**Figure 4:**
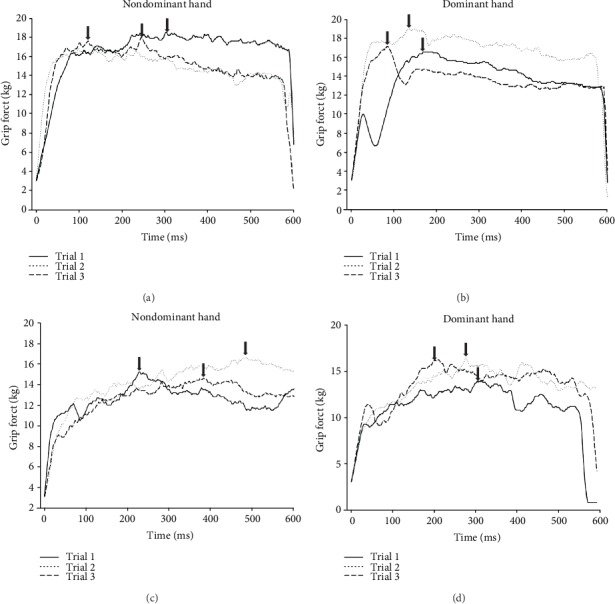
Representative plot of maximal hand-grip force measurements using a digital hand-held dynamometer in healthy young (a, b) and elderly (c, d) adults. The arrows indicate the peak value of the maximal hand-grip force.

According to the Bland-Altman plots, the mean bias between dynamometers was 13 kg with 1~25-kg limits of agreement in the dominant hand and a bias of 10 kg with 2~19-kg limits of agreement in the nondominant hand for all participants (Figure 1). The MicroFET3 dynamometer recorded lower values of grip force than the Jamar by 49.9%~57%; however, a significantly strong correlation was found between the Jamar and MicroFET3 dynamometers for both the dominant and nondominant hands for the young group (*r* = 0.76 and 0.80, respectively), elderly group (*r* = 0.72 and 0.72, respectively), and all participants (*r* = 0.82 and 0.84, respectively) (Table 2). A scatter-plot diagram revealed a formula to convert MicroFET3 dynamometer values to equivalent Jamar values for each hand as shown in Figure 2. A two-way ANOVA showed a significant interaction between dynamometers and groups in the dominant hand (*p* = 0.01), nondominant hand (*p* = 0.006), and both hands (*p* = 0.007), with a greater grip force required in the Jamar than in the MicroFET3 dynamometer and a greater amount of maximal voluntary grip-force contraction in young adults than in elderly adults for both the dominant and nondominant hands (Figure 3). Additionally, we also found that the maximal hand-grip force values generated in the dominant and nondominant hand by the elderly group were slower and more inconsistent than those of the young group when using the MicroFET3 dynamometer (Figure 4).

## 4. Discussion

This study indicated that hand-grip measurements, acquired using the MicroFET3 dynamometer, strongly correlated with the same measurements made by the Jamar dynamometer, in both hands, and for young and elderly adults. Grip-force measurements with the MicroFET3 dynamometer can be confidently converted to Jamar values using the formulae developed in this study. Age-related changes in maximum grip-force performance and grip strength control were found among elderly people.

### 4.1. Grip-Force Measurements and Development of Formulae for Converting MicroFET3 Dynamometer Force Values to Jamar Values

The Jamar dynamometer is one of the most commonly used measurement tools for measuring grip strength in clinical evaluations. Previous studies showed that nonpneumatic dynamometers such as the Baseline [23] and Dexter [24] had strong correlations with the Jamar dynamometer, and similar results were found in this study. However, grip forces measured by the MicroFET3 dynamometer were lower than those of the Jamar, and the discrepancy between the two dynamometers was greater than that in a previous study in which the difference in grip force measured by Rolyan and Jamar dynamometers was approximately 0.05~0.73 kg [8]. There are several potential reasons that might have caused this phenomenon, including different types and physical arrangements of the dynamometers, muscle length, and hand position while testing, and the size of the hands of subjects. First, the Jamar dynamometer has an adjustable handle, and subjects in this study were instructed to place their fingers in a position which caused the force mainly to be applied by the middle phalanx of the fingers when pulling the handle toward the palm to generate the maximal grip force [25]. In contrast, we found that when subjects held the MicroFET3 dynamometer device, the fingers were placed in a position such that the force was primarily applied by the distal phalanx of the fingers, which produces a relatively weaker grip force, and this finding is similar to those of earlier studies [25, 26]. This indicated that the force produced with the handle at the center is larger than when the handle is at the extremes. Additionally, the physical configuration of different measurement devices may also influence a grip-force generation, including the material, form, surface, and weight, but these need to be further investigated in future studies. In addition to the different types of dynamometers affecting the generation of the grip force, the muscle length and positions of the hands and forearm can also impact muscle activity and influence grip-force generation when performing grip-force measurements [26, 27]. Furthermore, the size of the hand can also influence the performance of grip strength, because we found that female subjects with smaller hands reported that they had great difficulty holding the farthest position of the dynamometer, which may result in a lower grip force; a similar finding was also shown in an earlier study [25].

Another interesting finding in this study was that we developed formulae for converting MicroFET3 dynamometer values to Jamar values. In previous studies, grip strength measurement tools such as a sphygmomanometer (which measures force in mmHg) [28] and the Manugraphy system (which measures force in Newtons) [10] were also reported to be capable of being utilized through conversion formulae equal to the Jamar unit (which measures force in kilograms). This suggests that the measured values of the grip force cannot directly be interchanged between different dynamometers and the Jamar model. In contrast, our findings indicated that the MicroFET3 dynamometer values can confidently be converted to equivalent Jamar dynamometer values with the formulae developed in this study. Therefore, grip-force values obtained from the MicroFET3 dynamometer can be compared to data from previous studies or normative data, since the majority of grip strength data were measured with the Jamar dynamometer [29, 30].

### 4.2. Age-Related Decline in the Maximum Grip-Force Performance

In this study, we found an age-related decline in the maximum grip-force performance, and these findings were similar to those of previous studies [31, 32]. Grip force is considered representative of upper limb function, a major predictor of an elderly person's ability to perform activities of daily living [33], to be strongly associated with mobility and balance [7], and an indicator of a person's general health status [34]. An early study also indicated that the development of frailty appears to be highly related to grip strength [35]. The AWGS recommends a diagnosis of sarcopenia use the presence of a low muscle mass, low muscle strength, and low physical performance [3], with cutoff values of maximum grip force for people with sarcopenia of <26 kg for men and <18 kg for women [3]. In this study, elderly men and women revealed mean grip forces of 31 ± 4 and 18 ± 4 kg, respectively, and 51% of the elderly women had a dominant hand-grip force of <18 kg. Further analyses showed that females with lower hand-grip strength (<18 kg) were significantly older than the others (mean age of 72 ± 5 vs. 68 ± 5 years old). Recent studies have indicated that advancing age is associated with lower grip strength [36] and a higher prevalence of sarcopenia [37]. We surmised that approximately half of our female subjects matched at least one of the sarcopenia criteria and might have a higher likelihood of sarcopenia; however, other criteria, such as walking velocity and muscle mass, were not assessed in this study. Additionally, other factors such as gender, height, and weight might also affect the grip strength [38, 39]. Furthermore, this study also showed that maximal hand-grip force values generated in both hands by the elderly group were slower and more inconsistent than those of the young group using the MicroFET3 dynamometer. This phenomenon was not shown and has been little reported in previous studies because most therapists only evaluate the instantaneous maximal hand-grip strength of both hands using the Jamar dynamometer and indicate age-related changes in grip-force performance. In humans, the neuromuscular and sensory systems are involved in maximal hand-grip generation. This involvement includes activation and neuron excitation in the primary motor cortex, primary sensory cortex, premotor cortex, the prefrontal cortex, supplementary motor area, and cerebellum in the brain. This activation results in action potentials being delivered to the neuromuscular junction, thereby facilitating muscle contractions within the hands via the peripheral nervous system [40]. However, these physiologic functions in neuromuscular and sensory systems can deteriorate with age and may result in slowing and inconsistencies in hand-grip generation in the hands of elderly adults. Other changes related to aging include atrophy of the motor cortical regions, reduction of the grey matter, reductions in the dendritic density of the brain [41–44], reduced nerve conduction, sensory system changes, and fewer and less sensitive somatosensory receptors in the skin, muscles, and joints [45–48]. De Dobbeleer et al. also reported similar grip dynamics during sustained grip efforts in healthy middle-aged individuals [49]. Gijzel et al. indicated that the slowing down and greater variance in dynamic time series measures were valid indicators of physical resilience. These findings help therapists understand the dynamic resilience of grip force control and hand function, enhance treatment selection, and improve the benefit to older people with frailty and multiple morbidities [50]. Therefore, we believe the delayed and inconsistent maximal hand-grip force generation data can provide valuable information for physical and occupational therapists and researchers to further understand age-related changes in muscle function and help in developing appropriate exercise programs to improve muscle function in elderly people.

Although this study pointed out that the MicroFET3 dynamometer can be an option for measuring the maximal grip force with good validity and grip-force values can be converted to Jamar values via the formulae we calculated, we found a limitation of the MicroFET3 dynamometer when measuring hand-grip strength. For instance, the MicroFET3 dynamometer has larger contact areas for generating more grip force without feeling pain, but it is still difficult to use by individuals with small hands such as children, because it does not have an adjustable handle, thereby decreasing the maximum grip strength generated. Additionally, as the MicroFET3 dynamometer could continually collect grip-force data and assess hand-grip force performance via the schematic diagrams, it showed evidence of the lower speed and inconsistent generation of maximal hand-grip force values, in both hands, in the elderly group compared to the young group. However, we did not statistically compare the differences in speed and maximal hand-grip force generation consistency between the young and elderly groups. We believe this valuable information would be very useful for physical and occupational therapists to apply this function to evaluate and analyze the quality of grip-force generation in patients with neurologic or orthopedic diseases, as it provides valuable information for clinical diagnoses of quality control of grip-force generation, by identifying patients with potential frailty, and developing appropriate rehabilitation interventions and health promotion programs for high-risk frail and disabled elderly adults.

## 5. Conclusions

In conclusion, this study indicated that the hand-grip strength measurement of the digital MicroFET3 dynamometer was highly correlated with that of the gold standard Jamar dynamometer and can be used to measure the hand-grip force, and the developed confidently conversion formulae can be used to enhance clinical applications of grip-force measurements of MicroFET3 dynamometer scores and compare them with the Jamar standard. The digital MicroFET3 dynamometer not only indicated age-related declines in the maximum grip-force performance but also revealed slower and more inconsistent maximal hand-grip strength generation by the elderly than the young group.

## Figures and Tables

**Table 1:** Demographic data of the young and elderly groups.

	Young group (*n* = 65)	Elderly group (*n* = 50)
Age (years)	23.3 ± 4.5	69.5 ± 5.8
Sex (male, *n*)	36 (55%)	3 (6%)
Dominant hand (right, *n*)	60 (92%)	49 (98%)
Height (cm)	168.9 ± 8.6	154.9 ± 22.2
Weight (kg)	63.9 ± 12.3	59.2 ± 17.2^∗^
MMSE (score)	—	27.7 ± 3.1

Values are presented as the mean ± standard deviation; MMSE: Mini-Mental Status Examination. ∗*p* < 0.05.

**Table 2:** Correlation coefficient *r* values between the Jamar and MicroFET3 dynamometers.

	Young group		Elderly group		All participants	
	*r* value	*p* value	*r* value	*p* value	*r* value	*p* value
Dominant hand	0.76	2.80 × 10^−13∗∗^	0.72	3.31 × 10^−9∗∗^	0.82	4.14 × 10^−29∗∗^
Nondominant hand	0.80	2.66 × 10^−15∗∗^	0.72	4.10 × 10^−9∗∗^	0.84	2.87 × 10^−32∗∗^

∗*p* < 0.05, ∗∗*p* < 0.001.

## Data Availability

The data used to support the findings of this study are available from the corresponding author upon request.
